# The efficacy-effectiveness distinction in trials of alcohol brief intervention

**DOI:** 10.1186/1940-0640-9-13

**Published:** 2014-08-18

**Authors:** Nick Heather

**Affiliations:** 1 Department of Psychology, Faculty of Health & Social Sciences, Northumberland Building, Northumbria University, Newcastle upon Tyne NE1 8ST, UK

**Keywords:** Alcohol-related harm, Brief interventions, Efficacy trials, Effectiveness trials, Meta-analyses

## Abstract

Three recent sets of null findings from trials of alcohol brief intervention (BI) have been disappointing to those who wish to see a reduction in alcohol-related harm through the widespread dissemination of BI. Saitz (7) has suggested that these null findings result from a failure to translate the effects of BI seen in efficacy trials, which are thought to contribute mainly to the beneficial effects of BI shown in meta-analyses, to effectiveness trials conducted in real-world clinical practice. The present article aims to: (i) clarify the meaning of the terms “efficacy” and “effectiveness” and other related concepts; (ii) review the method and findings on efficacy-effectiveness measurement in the 2007 Cochrane Review by Kaner and colleagues; and (iii) make suggestions for further research in this area. Conclusions are: 1) to avoid further confusion, terms such as “efficacy trial”, “effectiveness trial”, “clinical representativeness”, etc. should be clearly defined and carefully used; 2) applications of BI to novel settings should begin with foundational research and developmental studies, followed by efficacy trials, and political pressures for quick results from premature effectiveness trials should be resisted; 3) clear criteria are available in the literature to guide progress from efficacy research, through effectiveness research, to dissemination in practice; 4) to properly interpret null findings from effectiveness studies, it is necessary to ensure that interventions are delivered as intended; 5) in future meta-analyses of alcohol BI trials, more attention should be paid to the development and application of a psychometrically robust scale to measure efficacy-effectiveness or clinical representativeness; 6) the null findings under consideration cannot be firmly attributed to a failure to translate effects from efficacy trials to real-world practice, because it is possible that the majority of trials included in meta-analyses on which the evidence for the beneficial effects of alcohol BI was based tended to be effectiveness rather than efficacy trials; and 7) a hypothesis to explain the null findings in question is that they are due to lack of fidelity in the implementation of BI in large, organizationally complex, cluster randomized trials.

## Introduction

There recently have been three disappointing sets of findings from randomized controlled trials (RCTs) of alcohol brief intervention (BI) in primary care. From the standpoint of science, no findings are disappointing if they are an accurate reflection of reality; however, because these are all null findings, they are considered disappointing to those who wish to see a substantial reduction in alcohol-related harm through the widespread delivery of BI in routine practice. The fact that all three trials were located in primary health care, long considered the most promising setting for the delivery of alcohol BI [1], adds to this sense of disappointment.

In chronological order, the first findings were from a cluster RCT of a tailored, multifaceted improvement program in facilitating the implementation of BI by general medical practitioners (GPs) in The Netherlands (van Beurden et al.) [2]. The improvement program consisted of a range of activities aimed at GPs, their organizations, and their patients. It took advantage of best evidence on how best to encourage GPs to deliver BI and the extensive experience of several of the investigators in this area. It represented, in short, the best chance for success in encouraging GPs to become involved in the delivery of alcohol BI. Unfortunately, the authors concluded that their program “failed to show an effect and proved difficult to implement” (p. 1601) and that, “there remains little evidence to support the use of such an intensive implementation program to improve the management of harmful and hazardous alcohol consumption in primary care” (p. 1601).

The second set of findings came also from a cluster RCT of a training program for GPs in Wales [3], but the training in this case was for the delivery of brief behavior change counseling for multiple lifestyle behaviors (smoking, lack of exercise, unhealthy eating, and excessive alcohol consumption), so the main outcome measure was changes in patient rather than GP behavior. The BI training program, known as PRE-EMPT, was based on motivational interviewing [4] and its effects were compared with delayed training. Three months after a BI, there were no differences between groups in the proportion of patients reporting beneficial changes in at least one of the four risky behaviors, including excessive drinking. The conclusion was that training GPs in behavior change counseling had no effect on patient self-reported behavior change.

The third null findings came from the primary care arm of the SIPS (Screening and Intervention Programme for Sensible Drinking) project [5], a cluster RCT in England involving three conditions: (i) a control group given a patient information leaflet (PIL); (ii) a group given the PIL plus 5 minutes of structured brief advice, and (iii) a group given the PIL, brief advice, and 20 minutes of brief lifestyle counseling. At both 6- and 12-month follow-up, there were no differences between groups in the proportion of patients who had reduced their score on the Alcohol Use Disorders Identification Test (AUDIT) [6] from above to below the recommended cut point; i.e., indicating a beneficial change. The authors’ conclusion was that “… evidence that brief advice or brief lifestyle counseling provided additional benefit in reducing hazardous or harmful drinking compared with the patient information leaflet was lacking” (p. 2).

It is important to note that, given the large sample sizes in all three studies discussed here, lack of statistical power is unlikely to be the reason for null findings.

### An exchange of views

Because of their relevance to present concerns, comments by Richard Saitz on the SIPS findings [7] are worth quoting at length:

“Particularly given the robust findings from systematic reviews that favor brief intervention … when compared to no brief intervention in efficacy trials …, the conclusion most consistent with these data is that, even when great efforts are made to implement SBI (screening and brief intervention) in real-world clinical care (e.g., with less external researcher support), the effects seen in efficacy studies do not translate into effective interventions in practice.”

Saitz continues:

“And the effect sizes in efficacy studies, while large from a public health perspective, are small enough (e.g., three fewer drinks per week) that they could easily be erased when SBI is not implemented in practice exactly like it was in those studies….Yet alcohol SBI can only reach its potential if the effects seen in efficacy studies can be achieved in real-world practice. Kaner et al.’s [8] systematic review suggested that the practice was similarly effective in trials in which SBI implementation looked more like it would in clinical practice and less like research implementation, but none of those studies came close to being pragmatic trials like SIPS, so they couldn’t really inform that question.” (citation added)

Saitz observed that the SIPS trial was one of the few pragmatic implementation studies of alcohol SBI and that another trial, the van Beurden et al. trial [2] mentioned above, had an even more disappointing result. The overall conclusion was that “… researchers and educators should turn their attention to how to implement alcohol screening and brief intervention in clinical practice in a way that retains the efficacy seen in clinical trials.” Saitz here has put his finger on the most pressing challenge facing the alcohol BI field at the present time, a challenge that concerns the crucial distinction between efficacy and effectiveness research.

In their reply to Saitz, the SIPS investigators [9] wrote as follows:

“In contrast to Professor Saitz, we feel that the brief intervention evidence base to date has indicated … a growing preponderance of effectiveness rather than efficacy trials. (In the Cochrane Review) … the majority of studies … were judged to be clinically relevant effectiveness trials (with high external validity) rather than ideal-world efficacy trials (with high internal validity). In a field that has evolved for over 25 years, it is to be expected that evaluations have increasingly reflected the variability and constraints of real-world primary care.”

Kaner and colleagues also pointed to the difference between the SIPS trial, in which the aim was to evaluate the impact of SBI on patients’ drinking outcomes, and the van Beurden trial, which was a service-delivery trial to evaluate the impact of an intensive, multifaceted improvement program on GPs’ management of alcohol problems.

### Aims of this article

In this exchange of views, then, there appears to be disagreement and possibly some confusion over the meaning and applicability of the terms “efficacy”, “effectiveness”, “implementation, and “pragmatic” trials. There are also different views on whether the studies included in the Kaner et al. Cochrane Review [8] were primarily efficacy or effectiveness trials. With these issues in mind, the present article has three aims:

I. To clarify the meaning of the terms efficacy trial, effectiveness trial, and other related concepts, and to try to dispel some of the confusion surrounding these terms;

II. To review the method and findings on efficacy-effectiveness measurement in the Kaner et al. Cochrane Review [8];

III. To make suggestions for further research concerning the efficacy-effectiveness distinction.

### Explanatory versus pragmatic trials

In the current literature on alcohol BI, the terms “effectiveness trial” and “pragmatic trial” seem to be used synonymously. It is, of course, perfectly legitimate to use the word “pragmatic” in its ordinary language sense and, in this way perhaps, as the same in meaning to “effectiveness.” (The meaning of effectiveness trial will be considered below). However, the term pragmatic trial does have a more technical meaning deriving from a paper in the early literature by two French authors [10].

In this usage, explanatory trials are primarily concerned with understanding*,* whereas pragmatic trials are concerned primarily with decision*.* Thus, in a pragmatic trial, treatments are compared “under the conditions in which they would be applied in practice” (p. 638). A recent example is the United Kingdom Alcohol Treatment Trial (UKATT) [11] in which the intensity and therapeutic methods of the two forms of treatment being compared were deliberately confounded in the design (eight sessions of Social Behavior and Network Therapy versus three sessions of Motivational Enhancement Therapy, both over 12 weeks). This was because the aim of the trial was to determine which of two treatments was the more effective and/or cost-effective in the form in which it was intended to be delivered and in order to inform a decision as to which should be rolled out in routine practice in the UK National Health Service. If this had been an explanatory trial, it would have been necessary to control for either intensity or treatment type so that the effect of the other on outcomes could be independently assessed; but this was not necessary in a pragmatic trial. The explanatory-pragmatic distinction is clearly similar in some ways to efficacy-effectiveness, but it has special implications for the aims and design of a trial.

### The seminal work of Brian Flay

Flay [12] published his seminal paper on the efficacy-effectiveness distinction in 1986, certainly the first in the area of substance use disorders and possibly in public health in general. Flay credits Cochrane [13] in 1971 with first making the distinction in question, though the potential for confusion here is illustrated by the fact that Cochrane used “effectiveness” and “efficiency” for Flay’s “efficacy” and “effectiveness.” Flay’s paper is concerned with health promotion and is illustrated by examples from smoking prevention, but it is highly relevant to research on alcohol BI.

Flay first provides general definitions:

● Efficacy trials provide tests of whether a technology, treatment, procedure, or program does more good than harm when delivered under optimum conditions.

● Effectiveness trials provide tests of whether a technology, treatment, procedure, or program does more good than harm when delivered under real-world conditions.

Note immediately that efficacy is necessary to but not sufficient for effectiveness (i.e., if a treatment is effective, it must be efficacious but, if it is efficacious, it need not necessarily be effective). Thus, if an effectiveness trial produces a null result, one cannot be sure without a preceding efficacy trial whether the null result is due to lack of efficacy or lack of effectiveness.

In somewhat more detail, an efficacy trial provides a test of (i) a well-specified standardized treatment/program that (ii) is made available in uniform fashion, within standardized contexts/setting, to a specified target group, which (iii) completely accepts, participates in, complies with, or adheres to the treatment/program as delivered. However, an intervention in a real-world setting will be effective only if an efficacious intervention is delivered/implemented in such a way as to be made available to an appropriate target clientele in a manner acceptable to them (i.e., that they will be receptive to, participate in, comply with, or adhere to). Thus, the observed effects, or lack thereof, of an intervention in an effectiveness trial may be due to one or more of the following: (i) the efficacy level of the evaluated intervention; (ii) the availability of the intervention to the target population; or (iii) the level of acceptance of (participation in, compliance with, or adherence to) the intervention by the target group.

However, there are two types of effectiveness trials: treatment effectiveness trials and implementation effectiveness trials. Relationships between three types of research – efficacy trials and the two types of effectiveness trials – are shown in Table 1, which illustrates the role of the two key variables, availability and acceptance, underlying the distinctions between them. An efficacy trial optimizes both availability and acceptance; a treatment effectiveness trial optimizes availability and leaves acceptability to vary; and an implementation effectiveness trial leaves both to vary.

**Table 1:** **Three levels of experimental assessment determined by variation in three factors (adapted from Flay**[12]**)**

**Level of experimental assessment**	**Program implementation**	**Availability**	**Acceptance**
Efficacy	Standardized	Optimized	Optimized
Treatment effectiveness	Efficacious	Optimized	Variable
Implementation effectiveness	Efficacious	Variable	Variable

A key difference between treatment and implementation effectiveness trials is that the main outcome variable in the former refers to some aspect of patient/client behavior, whereas the main outcome in the latter concerns the behavior of the practitioners who deliver the interventions. From this point of view, it is clear that SIPS [5] was a treatment effectiveness trial and the van Beurden et al. trial [2] was an implementation effectiveness study. Though the PRE-EMPT trial [3] was an examination of the effects of training, the implementation of the intervention was controlled and it was the acceptability of the intervention to clients that was left to vary. So, with the main outcome being changes in patient behavior at follow-up, this was a treatment effectiveness trial. In relation to the van Beurden trial [2] it seems that the null findings were due to the failure of the improvement program to motivate enough GPs to deliver BI, not to the failure of the intervention itself to affect patients’ behavior. It is therefore not relevant to the main issue under discussion – the comparison of effects between efficacy and treatment effectiveness trials.

For present purposes, the main thing missing from Flay’s discussion is a consideration of intervention fidelity; i.e., the extent to which the intervention is delivered as intended and as shown to have been efficacious in previous research. In Flay’s logic, it is simply assumed that, in both kinds of effectiveness trials, the intervention has been implemented as intended. So, the main difficulty in mounting a true effectiveness study in Flay’s terms is of ensuring that the intervention was delivered faithfully in its efficacious form. The gap between a BI protocol and its delivery in general practice has been described in the literature [14] and was first noted as a problem in the conduct of the earliest trial of alcohol BI in primary care [15]. Implementation fidelity is especially likely to be an issue in large, organizationally-complex, cluster randomized trials like SIPS [5] and PRE-EMPT [3].

### Phases of research in the development of preventive interventions

Based on his analysis of the efficacy-effectiveness distinction and experience with research on smoking cessation, Flay [12] proposed eight phases of research that should underlie the development of health promotion programs. Rather than show this particular sequence of phases of research, Table 2 shows a somewhat simpler sequence derived from Flay’s later collaboration with Harold Holder and colleagues on phases of alcohol problem prevention research [16]. In phase IV of this sequence, the distinction between treatment effectiveness trials and implementation effectiveness trials has been collapsed; but this is still highly relevant to the discussion here.

**Table 2:** **Phases of alcohol problem prevention research (from Holder et al., 1999**[16]**)**

I.	Foundational research: Basic studies to define and determine the prevalence of specific alcohol-involved problems, establish the causal factors that yield specific problems or increase the risk of a problem, and provide foundations for the development of effective preventive interventions.
II.	Developmental studies: Preliminary studies to develop and test new interventions or to assess the effectiveness of an existing intervention
III.	Efficacy studies: Rigorous studies (of maximised internal validity) of the intervention under optimal conditions with maximal implementation (availability or enforcement) and acceptance (participation or compliance)
IV.	Effectiveness studies: Studies of real-world effectiveness of preventive interventions with purposeful or natural variations.
V.	Diffusion studies: Studies of the effects of different levels or types of implementation or acceptance on effectiveness

In considering the contents of Table 2, and assuming they represent a logical, coherent, and desirable sequence of phases, the question arises to what extent the history of research on alcohol BI has conformed to it. It seems obvious to the present author that the answer to this question is “hardly at all.” For example, in the first-ever trial of alcohol BI in primary health care in Dundee, Scotland [17], the investigators were blissfully unaware of the need to begin by establishing efficacy and plunged directly into a test of the DRAMS BI package in everyday conditions of busy general medical practice. Later well-known trials [18-21] may have included more features of efficacy research, for example, by using artificial methods of screening and identifying risky drinkers rather than leaving this to routine practice as in the DRAMS trial [17], but interventions were still conducted in real-world conditions of primary care.

A collection of studies that could lay some claim to being a logical progression of research was the WHO Collaborative Project on Identification and Management of Alcohol-related Problems in Primary Health Care [22]. (The WHO project was divided into four phases of research over 20 years, but these phases should not be confused with the phases of alcohol problem prevention research shown in Table 2.) This progression included: foundational research (see Table 2) in WHO Phase I, the development of a screening instrument, the AUDIT, specifically intended for use in primary health care [23]; a number of implementation effectiveness trials in different countries in WHO Phase III [24]; and a diffusion study in WHO Phase IV [25]. However, WHO Phase II [26] was again carried out in real-world conditions and did not show all the essential features of an efficacy trial.

The argument here is that the great majority of trials of alcohol BI in the literature have not, as Saitz [7] suggests, been efficacy trials, but treatment effectiveness trials. In Flay’s [12] terms, they were trials that have ensured the availability of the intervention by delivering a BI to all participants in a standard package but have left acceptability free to vary; to be an efficacy trial, it must be ensured that the BI has been accepted (complied with, adhered to) by selecting participants for the study among whom acceptance can be assumed, rather than participants encountered in routine practice among whom it cannot. This argument will be subjected to more objective scrutiny later in this article, when the relevant findings of the meta-analysis by Kaner and colleagues [8] will be described.

To anticipate one of the conclusions of this article, what are the implications of this argument, if accepted, for ongoing research on BI? As far as the primary care setting is concerned, we are, in the popular phrase, where we are; there is no suggestion here of turning the clock back to carry out efficacy research. The efficacy of BI in primary care can be inferred from the fact that it has emerged with evidence of beneficial effects from a long succession of systematic reviews and meta-analyses [27]. It is difficult to believe that this situation could have arisen without BI being efficacious, although the conclusion might have been reached earlier if treatment effectiveness research had been preceded by efficacy trials.

To implement BI research in the increasing number of settings in which it is desired (e.g., dentistry, colonoscopy, needle and syringe exchange programs, pharmacies, a range of criminal justice and educational settings) and where there is as yet no evidence base to speak of, there is an opportunity to conduct studies properly by beginning with foundational research where necessary, developmental studies (e.g., to discover the ways in which BI should be adjusted to the needs and characteristics of the recipients and the exigencies of the particular settings), and efficacy trials under carefully controlled conditions of intervention availability and participant compliance. To do so, rather than going directly to effectiveness trials, will eventually save time and money. Unfortunately, there are often pressures from funders and other stakeholders for rapid results and quick justifications for policies aimed at implementing BI in as many plausible settings as possible. These political pressures should be resisted in the interests of real progress on how to implement alcohol BI to reduce harm.

### Flay’s later work

Flay has continued his work in this field of study and in 2005 produced a report with colleagues based on the deliberations of a committee established by the Society for Prevention Research in the United States and charged with establishing standards for identifying effective prevention programs and policies [28]. This report concluded that an efficacious intervention will have been tested in at least two rigorous trials that: (i) involved defined samples from defined populations; (ii) used psychometrically sound measures and data collection procedures; (iii) analyzed their data with rigorous statistical approaches; (iv) showed consistent positive effects (without serious iatrogenic effects); and (v) reported at least one significant long-term follow-up.

An effective intervention will not only meet all standards for efficacious interventions but also will have: (i) manuals, appropriate training, and technical support available to allow third parties to adopt and implement the intervention; (ii) been evaluated under real-world conditions in studies that include sound measurement at the level of implementation and engagement of the target population (in both the intervention and control conditions); (iii) indicated the practical importance of the intervention outcome effects; and (iv) clearly demonstrated to whom the intervention findings can be generalized.

Lastly, an intervention recognized as ready for broad dissemination will not only meet all standards for efficacious and effective interventions but will also provide: (i) evidence of the ability to go to scale; (ii) clear cost information; and (iii) monitoring and evaluation tools so that adopting agencies can monitor or evaluate how well the intervention works in their settings. All these and the preceding recommendations from Flay’s later work are directly relevant to research on alcohol BI.

### Analysis of the efficacy-effectiveness dimension in the 2007 Cochrane systematic review

As noted above, the issue of efficacy versus effectiveness of research in the alcohol BI field was subjected to empirical scrutiny in a highly influential systematic review and meta-analysis by Kaner and colleagues [8]; this has already been referred to above in summarizing the exchange of views between Saitz [7] and the SIPS investigators [9] following the publication of the primary care arm of the SIPS trial [5].

A subgroup analysis was undertaken to assess the impact of brief interventions in efficacy (ideal-world) and effectiveness (real-world) trials using a coding scale adapted from the work of Shadish and colleagues [29]. Note immediately the assumption here that the distinction between efficacy and effectiveness forms a multidimensional domain and can be represented as continuous variation along a scale. Shadish et al. had used 10 codes to distinguish between ideal and real-world trials of psychological therapy in general and had applied these codes to 60 trials to generate a scale score for each, which they then related to the trials’ reported effect sizes. The conclusion of this exercise, incidentally, was that “… psychological therapies are robustly effective across conditions that range from research-oriented to clinically representative” (p. 522).

The eight coding items that were adapted from the codes of Shadish and colleagues and were applied to all 21 RCTs included in the Cochrane Review are shown in Table 3. The four items thought on *a priori* grounds to have greater relevance to the effectiveness of alcohol BI scored 2/0, and the four items with less apparent relevance scored 1/0, giving a range of scores from zero to 12. If an item appeared to be partially clinically representative on any item, then a midpoint score was given (either 1 or 0.5, as applicable). Similarly, if authors did not report data relating to a particular item, the midpoint score was used. Each trial was independently coded by two authors. If there was disagreement concerning a coding, this was resolved through discussion in order to gain consensus.

**Table 3:** **Eight coding items used to form a scale of efficacy-effectiveness (or clinical representativeness) (adapted from Kaner et al. **[8]**)**

***Patients and problems***	***Therapeutic flexibility***
2 = clinically representative subjects initially present with a typically wide range of problems via self-referral or invitation for a health check.	1 = clinically representativeness allows professional judgement in how an intervention is delivered (e.g., freedom to focus on particular issues according to patient need).
0 = research representative subjects may be paid patients, researcher-solicited volunteers (e.g., via advertisement) or referrals from specialist services.	0 = research representativeness would be strict adherence to a prescribed protocol or script that does not allow for variability in practice.
***Practice context***	***Pre-therapy training***
2 = clinically representative is a community-based setting in which a range of clinical services are usually provided to patients.	1 = clinically representative training in intervention procedures occurs according to typical CPD/CME procedures (e.g., outreach visits, seminars, one-off training days).
0 = research representative is a setting in which the research function clearly dominates any clinical one (e.g., clinic at a university or hospital).	0 = research representative training is unusually intensive or requiring of atypical levels of interest or motivation (e.g., prolonged or intensive courses, formal qualification).
***Practitioners and therapists***	***Intervention support***
2 = clinically representative practitioners are practising doctors, nurses, and qualified therapists who earn their main living by providing health services in primary care.	1 = clinically representative support occurs within standard practice resources (e.g., colleague assistance with screening, IT flagging).
0 = research representative practitioners are nonclinicians or clinicians in training who are contracted to deliver interventions for the purposes of the study.	0 = research representative support would not typically be available (e.g., researcher help to flag notes, extra staff for period of the trial).
***Intervention content***	***Intervention monitoring***
2 = clinically representative intervention fits with current practice in terms of timing, content or style (e.g., 5–15 minutes for a GP; 20–30 minutes for a nurse or initial screening accompanied by a return visit for brief intervention).	1 = clinically representative monitoring of intervention delivery does not interfere with practitioners’ behaviour or their relationship with patients.
0 = research representative treatment would not normally occur in routine practice (e.g., unusually long consultations).	0 = research representative monitoring would be direct observation of therapist behaviour or ongoing/immediate feedback to practitioners after each session.

**Figure 1:**
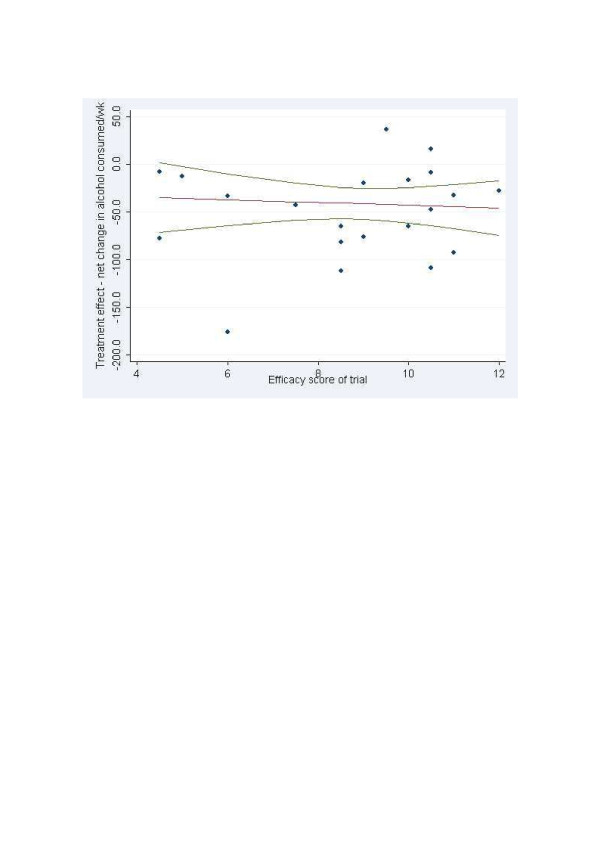
**Estimated treatment effect versus effectiveness/efficacy score.** The lines show the predicted metaregression line and its 95%CI. (from Kaner et al. [8,24]). NB. Increasing scores on the abscissa indicate greater effectiveness.

Figure 1 shows the estimated treatment (i.e., intervention) effect of a trial on the ordinate, plotted against its score on the efficacy-effectiveness dimension on the abscissa (with increasing scores indicating greater effectiveness.) It will be seen that scores tended toward the right-hand of the scale; i.e., in the direction of greater scores towards the effectiveness end of the spectrum, with a median of 9 and an inter-quartile range of 8–10.5. Scores ranged from 4.5 [30,31] to 12 [32].For the purpose of further subgroup analysis, a binary variable was created with a cut-point at the median, with those trials to the left of the median in Figure 1 being classified as efficacy trials, while those to the right were classified as effectiveness trials, remembering that these labels are relative because most trials had scores towards the effectiveness end of the spectrum. Comparing these two groups, there was no significant difference between trials classified as effectiveness and efficacy trials in the effect of brief intervention on the quantity of alcohol consumed; further, meta-regression showed no significant relationship between the estimated treatment effect and the efficacy score of the trial. Inspection of Figure 1 confirms that there is little if any relationship between a trial’s score on the efficacy-effectiveness dimension and its effect size. The authors do concede the possibility that this lack of relationship may indicate insensitivity in the classification tool. Nevertheless, they conclude:

“In the field of brief alcohol intervention, there has been a growing view that most of the trials to date have been tightly controlled efficacy studies and not particularly representative of routine clinical practice (Babor et al., 2006) [33]. …..within the context of trial-based evaluation, we feel that the current body of brief alcohol intervention research is applicable to clinical practice. Previous trials have fallen on a continuum from efficacy to effectiveness trials, and the lack of significant difference in outcomes on this dimension suggests that this body of work can inform routine practice” (p. 19).

In addition to the reference to the work of Babor and colleagues [33] in the quotation above, if it had been available to them at the time, the authors of the Cochrane Review could have cited the letter by Saitz in the *British Medical Journal*[7] commenting on the SIPS findings. Thus, an implication of the results of the subgroup analysis summarized above is that Saitz is mistaken in believing that the majority of BI trials in the literature are efficacy trials or that effectiveness trials tend to have weaker effects on treatment outcome than efficacy trials (or, at least, those tending more towards the efficacy end of the spectrum). Hence, if the subgroup analysis in question is valid, the null findings of the trials described at the outset of this article cannot be attributed to the difficulty in translating effects of BI seen in efficacy trials to effectiveness trials.

Before this conclusion and its implications are fully accepted, however, it must be recognized that the subgroup analysis in question can be criticized on technical grounds. First, although it was reported that trials were independently coded by two authors, there was no mention of pilot work to establish the reliability of the codings, and no measure of agreement between coders was reported. Perhaps more damagingly, no psychometrics were carried out on the efficacy-effectiveness scale; e.g., principal components analysis to test for unidimensionality and then deletion of coding items that lowered Cronbach’s alpha. Lastly, there were no comparisons between the efficacy and effectiveness trial groups on effect sizes for individual scale items. This possibility was recognized by the authors when they wrote: “It is possible that the treatment effect may be related to some of the individual factors, which were combined in the efficacy score. However, we did not investigate this as it would have been a *post hoc* analysis, not specified in the protocol” [8] (p. 17).

As this suggests, an analysis of this kind could be planned for inclusion in any future meta-analysis of BI RCTs. More generally, the subgroup analysis could be repeated in a future meta-analysis without the flaws identified above. Whether this would make any difference to the general conclusion reached regarding the essential effectiveness of most trials in the literature is an interesting empirical question.

One last point should be made about the subgroup analysis in the Cochrane Review. In discussing the interpretation of their coding scheme and scale, Shadish et al. [29] specifically reject the idea that the degree of clinical representativeness of trials they aim to measure can be equated with efficacy-effectiveness based on internal versus external validity. To do so, they say, results in an oversimplification because, in classic discussions of internal-external validity [34,35], the crucial methodological features for high internal validity are random assignment and the minimization of attrition. It is clearly possible for a trial that is clinically representative to meet these two criteria and thus to be both clinically representative and internally valid. This persuasive argument suggests that the dimension analyzed by Kaner and colleagues in the 2007 Cochrane Review [8] was clinical representativeness, not efficacy-effectiveness based on relative degrees of internal and external validity.

### Other relevant scales

Before concluding this review of the efficacy-effectiveness dimension, it may be useful to note the publication of two other scales with relevance to the issue. In the first, Gartlehner and colleagues [36] developed and tested a simple instrument based on seven criteria of study design to distinguish effectiveness from efficacy trials. These authors began by noting that no validated definition of ‘effectiveness study’ exists. They carried out a search for existing scales to measure the efficacy-effectiveness dimension but found none. (They obviously missed the scale developed by Kaner and colleagues [8] and noted above, perhaps because it was buried in a longer Cochrane Review; this suggests the need to make the issue of efficacy-effectiveness or the clinical representativeness of trials the topic of separate publications.) It should be stressed, however, that the efficacy-effectiveness scale developed in this project, based as it was on research design and influenced largely by a conventional understanding of internal and external validity, was very different from the scale used by Kaner and colleagues [8], suggesting again that the latter would be better termed “clinical representativeness.”

The second novel scale was developed by Thorpe and colleagues [37] and was intended to reflect the explanatory-pragmatic dimension. However, these authors’ understanding of an explanatory trial differed from that of the originators of this term; i.e., as describing trials that were designed to test causal hypotheses regarding the way an intervention exerts its effect [10], and was based rather on trials that aimed to provide an answer to the question, “Can this intervention work under ideal conditions?” (as opposed to pragmatic trials, which tried to answer the question, “Does this intervention work under usual conditions?”). This unhelpful change in terminology illustrates again how easily confusion can be introduced into this field of study by careless use of terms. Nevertheless, this scale, which differs again from the two considered above [8,37], may be useful to researchers. Indeed, in future meta-analyses of alcohol BI trials, two or even all three of the scales considered here, measuring as they do somewhat different aspects of ideal-world versus real-world trials, could be used and relationships between them explored.

## Conclusions

The following are summaries of conclusions in the preceding text:

1. There is considerable confusion and inconsistency in the literature regarding such terms as efficacy trial, effectiveness trial, explanatory trial, pragmatic trial, and clinical representativeness. In the future, these terms should be clearly defined and carefully used.

2. It is a mistake to go straight to effectiveness trials for new forms of alcohol BI intended for different populations in different settings where the evidence base is thin or nonexistent. The development and testing of new applications of BI should begin with foundational research and developmental studies, followed by efficacy trials, before large-scale effectiveness trials are mounted. Political pressures for quick results from premature effectiveness trials should be resisted.

3. Clear criteria are available in the literature to guide progress in movement from efficacy research, through effectiveness research, to dissemination in practice.

4. To properly interpret the findings of effectiveness studies, especially null findings, it is necessary to ensure that interventions are delivered as intended and as found efficacious or effective in previous research.

5. In future meta-analyses of alcohol BI trials, more attention should be paid to the development and application of a scale to measure efficacy-effectiveness or clinical representativeness, including: theory-based scale construction; inter-rater reliability testing and reporting; psychometric scale refinement; and publication as a topic of interest in its own right.

6. In relation to the three disappointing findings with which this article began:

a. The null findings of the van Beurden et al. trial [2] are not relevant to the issue of translating efficacy into treatment effectiveness because they represent a failure of implementation effectiveness. They reinforce strongly what is already known – that it is extremely difficult to get health professionals to deliver alcohol BI.

b. The null findings of the SIPS trial [5] cannot be firmly attributed to a failure to translate effects from efficacy trials to real-world practice because it is possible that the majority of trials included in meta-analyses on which the evidence for the beneficial effects of alcohol BI is based tended to be effectiveness trials rather than efficacy trials (although the validity of this conclusion should be more rigorously tested in future meta-analyses of alcohol BI).

c. A leading hypothesis to explain the null findings of the SIPS [5] and PRE-EMPT [3] trials is that they are due to lack of fidelity in the implementation of BI in large, complex, cluster randomized trials.

## Competing interests

The author was one of the authors of the 2007 Cochrane Review and a Principal Investigator of the SIPS trial, both of which are mentioned prominently in this article. Other than those, he has no competing interests to declare.

This article is based on a presentation at the 10^th^ Annual Conference of the International Network on Brief Interventions for Alcohol and Other Drugs (INEBRIA), Rome, Italy, 20^th^ September, 2013.
